# Understanding the Factors Influencing Patient E-Health Literacy in Online Health Communities (OHCs): A Social Cognitive Theory Perspective

**DOI:** 10.3390/ijerph16142455

**Published:** 2019-07-10

**Authors:** Junjie Zhou, Tingting Fan

**Affiliations:** Department of Business Administration, Business School, Shantou University, No.243 Daxue Road, Shantou 515063, Guangdong, China

**Keywords:** e-health literacy, online health communities, social cognitive theory

## Abstract

Although online health communities (OHCs) are increasingly popular in public health promotion, few studies have explored the factors influencing patient e-health literacy in OHCs. This paper aims to address the above gap. Based on social cognitive theory, we identified one behavioral factor (i.e., health knowledge seeking) and one social environmental factor (i.e., social interaction ties) and proposed that both health knowledge seeking and social interaction ties directly influence patient e-health literacy; in addition, social interaction ties positively moderate the effect of health knowledge seeking on patient e-health literacy. We collected 333 valid data points and verified our three hypotheses. The empirical results provide two crucial findings. First, both health knowledge seeking and social interaction ties positively influence patient e-health literacy in OHCs. Second, social interaction ties positively moderate the effect of health knowledge seeking on patient e-health literacy. These findings firstly contribute to public health literature by exploring the mechanism of how different factors influence patient e-health literacy in OHCs and further contribute to e-health literacy literature by verifying the impact of social environmental factors.

## 1. Introduction

### 1.1. Online Health Communities

Online health communities (OHCs), as a specific application of information and communication technologies (ICTs), have recently become a crucial platform for individuals to conduct health-related activities [1,2,3]. The OHC is a specific type of virtual community (VC). It enables and facilitates “social networking, participation, apomediation, collaboration, and openness within and between different health-related stakeholders” (i.e., health care consumers, caregivers, patients, health professionals, and biomedical researchers) [4,5]. In OHCs, health care consumers and patients can exchange health knowledge and social support [6,7,8] and/or make online appointments with health professionals [9,10,11]. Health caregivers and professionals can use OHCs to deliver health information [12,13] and/or conduct health-related education [14,15]. These advantages make OHCs important places to manage public health activities. 

As OHCs have become popular, more and more health-related stakeholders have begun to use OHCs to promote personal e-health literacy. E-health literacy sources from health literacy and refers to “the ability to seek, find, understand and appraise health information from electronic sources and apply knowledge gained to addressing or solving a health problem” [16]. E-health literacy is a behavioral health outcome that can be improved by different interventions (e.g., OHCs) [17,18,19]. Because of the user-generated contents, OHCs are like a health knowledge reservoir that is full of health-related resources shared by different health-related stakeholders [5,20,21]. Consumers can seek and find useful health knowledge in OHCs and use them to tackle their health issues or check up on their health status [22,23]. These activities are helpful for patients to conduct health self-management activities and empower themselves [24,25].

### 1.2. E-Health Literacy

Scholars place emphases on e-health literacy because e-health literacy directly reflects individuals’ ability to use online health-related resources and in turn achieve better health outcomes [16,24]. Exploring the ways of promoting e-health literacy can help consumers better utilize the health information on the Internet. During the past decades, scholars have also explored the potential factors influencing individual e-health literacy (summarized in Table 1). 

Based on the prior studies shown in Table 1, we can draw two major conclusions. First, prior studies mainly focused on the effect of Internet use; however, few studies investigated the effect of OHCs that act as a specific intervention on e-health literacy. Future studies should provide more positive evidence to support the roles of OHCs in promoting individual e-health literacy [18]. Second, prior studies mainly focused on the effect of demographic factors (e.g., age, gender, and education) [2,26,31,32,33]; however, a recent review study has also stressed the importance of accessible media environments and contextual spheres [34]. 

Although there are many studies that have addressed e-health literacy in OHCs, few studies have investigated the factors influencing patient e-health literacy in OHCs. OHCs and other social media are crucial interventions to eliminate the digital divide and promote public health [3,4,14,17,18,24,35]. OHCs provide vulnerable populations with an effective toolkit to access health information and services and give them great promise to influence behavioral health outcomes via their interactivity features and user-generated content mechanism [36,37,38]. E-health literacy, as a personal health outcome state, is a result of both personal determinants (i.e., personal behaviors and cognitive factors) and socio-structural determinants (i.e., social environmental factors) of health [39,40,41]. However, there is a paucity of studies that have investigated the specific mechanism of how personal determinants and social environmental factors influence patient e-health literacy in OHCs [34]. This paper aims to address the above gap. We focus on the following question:

How do patient OHC participatory behavior and OHC social environmental factors influence a patient’s e-health literacy?

### 1.3. Social Cognitive Theory

We adopt social cognitive theory (SCT) as the theoretical foundation to address the above question. SCT is a widely accepted theory in explaining individual behaviors. SCT is triadic reciprocal determinism in which individual behaviors are a result of three factors: environment, person, and behavior [42]. The person refers to personal individual cognitive factors; the environment includes both physical environment and social environment; and the behavior refers to a focal individual’s actions [42]. According to SCT, every two out of the three factors can interact with each other and then influence the third one; for example, human beliefs and cognitive competencies can be developed and modified by personal behaviors and structures within the environment [42]. 

During the past several decades, SCT has been widely used in explaining different individual behaviors (e.g., health knowledge sharing behaviors in VCs [43] and adoption intention of telehealth systems [44]) and in explaining personal cognition change (e.g., personal-computer-related technostress [45] and health outcomes [46]). Considering that health outcome change is not only a result of personal behaviors but is also under the influence of social systems [39,40,41], it is suitable to use SCT to explore the factors influencing patient e-health literacy in OHCs. 

### 1.4. Hypotheses and Research Model

Based on SCT and prior studies on e-health literacy, we chose patient health knowledge seeking as a personal behavior and social interaction ties as a social environmental factor in OHCs. Health knowledge seeking behaviors refer to a patient’s consumption of the health knowledge that is available in an OHC or their soliciting of answers or help from other members of the same OHC [47]. Social interaction ties refer to the strength of the relationships between different interaction parties, the amount of time spent, and the communication frequency among different OHC members, and they act as channels for information and resource flows [48]. Directed by SCT, we treated patient e-health literacy as a consequence of both individual behaviors (i.e., health knowledge seeking) and the social environment (i.e., social interaction ties) [39,40,41].

The user-generated content mechanism makes OHCs a health knowledge reservoir. With the facilitation of technologies, users dispersed in different places can freely access OHCs and collaboratively conduct health-related activities. For typical OHC users, seeking health knowledge is one of their basic targets [8,21,49]. They even can seek and exchange health knowledge, treatment experience, or personal information on embarrassing conditions or stigmatized illnesses [7,21,50]. These knowledge seeking behaviors are helpful for patients to manage self-education activities [17,18] and improve their e-health literacy [2,28]. We thus hypothesize the following:

**Hypothesis** **1.** Patient health knowledge seeking behaviors in OHCs positively influence their e-health literacy.

Besides personal health-related behaviors, the social environment around patients also influences their health-related outcomes [40,41]. Typical users also turn to OHCs for social support or companionship [4,6,7]. They look for patients like them, and they interact and communicate with them to conduct health-related activities. Such an interaction activity acts as an important part of the community sphere and in turn influences patient health outcomes (e.g., e-health literacy) [34]. We thus hypothesize the following:

**Hypothesis** **2.** Social interaction ties as an OHC social environmental factor positively influence patient e-health literacy.

Social interaction ties are crucial channels for information and resource flows [48]. In OHCs, these information and resource flows are completed via texts, pictures, and/or videos. However, compared with face-to-face communication, online communication channels such as texts, pictures, and/or videos belong to typical lean media and can only deliver limited information [51]. Social interaction ties can change the above situation. Stronger social interaction ties are powerful indicators of an active community sphere and harmonious relationships [48]. They provide users with more opportunities to conduct health-related activities, which are helpful for them to understand and use the knowledge that other members have shared and then obtain better health outcomes. We thus hypothesize the following:

**Hypothesis** **3.** Social interaction ties positively moderate the effect of health knowledge seeking behavior on patient e-health literacy; i.e., when social interaction ties are strong, the effect of health knowledge seeking behavior on patient e-health literacy will be high; when social interaction ties are weak, the effect of health knowledge seeking behavior on patient e-health literacy will be low.

**Figure 1:**
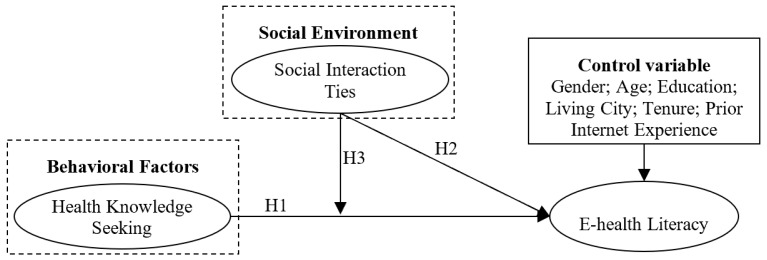
Hypotheses and research model. Note: H1, H2, and H3 respectively short for Hypothesis 1, Hypothesis 2, and Hypothesis 3.

Besides the above factors, patient gender, age, education level, living city, tenure in an OHC, and prior Internet experience might also influence patient e-health literacy. We treated these as control variables. Based on the above factors, we built a research model as shown in Figure 1.

As discussed above, this paper aims to explore how patient health knowledge seeking behaviors and their social interaction ties in OHCs influence their e-health literacy. We will examine their direct effects and interaction effect to address how OHC use influences patient e-health literacy.

## 2. Materials and Methods

### 2.1. Research Design

We adopted a survey approach. This study was approved by STU academic and ethical committees. We developed scales to estimate the three key constructs (i.e., health information seeking, social interaction ties, and e-health literacy). All scales for the three key constructs were adopted from prior studies and adapted to the OHC context. Health knowledge seeking had three items [49]; social interaction ties had four items [48]; and e-health literacy had eight items [16]. All items were scaled with a 5-point Likert scale with 1 representing “totally disagree” and 5 representing “totally agree”.

### 2.2. Data Collection

We developed a questionnaire composed of the above scales and demographic variables to collect data. The questionnaire was made via an online survey system. We asked the help of community administrators to allow us to post the questionnaire link in their community and finally were approved by four communities. The questionnaire was totally voluntary and anonymous. In order to improve the respondent rate, we provided 50 textbooks to randomly selected participants after the survey was over. The 50 textbooks are current popular books on how to preserve health and their prices were about 30 Chinese Yuan (about 4.3 dollars). 

We took several countermeasures to ensure high data quality. First, we added the question “have you ever used an OHC” to identify those actual OHC users. Because the questionnaire link is open and could be answered by anyone on the online survey system, we added the above question to identify the actual OHC users. If they answered “no”, the survey would end; otherwise, they would be required to write out the OHC name. Second, each IP address was allowed to answer only once to avoid repeated participants. Third, we added a reverse question to exclude careless respondents. If a respondent failed to notice the reverse question, the sample will be treated as an invalid one. The survey period lasted two months. There are 409 participants who have ever used an OHC, and 76 out of 409 respondents who had failed in answering the reverse question. We threw out the 76 respondents and finally obtained 333 valid data points. The descriptive statistics results are shown in Table 2.

### 2.3. Data Analysis

We conducted exploratory factor analysis (EFA, see Table 3) and confirmatory factor analysis (CFA, see Table 4) to estimate the measurement model. 

We used two methods to estimate convergent validity [47]. As shown in Table 3, all factor loadings in their respective constructs are greater than 0.7; in addition, all Cronbach’s α and composite reliability values are greater than 0.8. These indices suggest a good convergent validity.

We used two methods to estimate discriminant validity [47]. As shown in Table 3, all factor loadings in their respective constructs are significantly greater than the values in their irrespective constructs. In addition, the values in diagonal lines (i.e., average variance extraction (AVE) square root) in Table 4 are greater than the respective values in non-diagonal lines (i.e., correlation value). These indices suggest a good discriminant validity.

We further estimated the model fit. As shown in Table 5, the values of χ2/df, GFI (Goodness-of-Fit Index), AGFI (Adjusted GFI), NFI (Normed Fit Index), CFI (Comparative Fit Index), and RMSEA (Root-Mean-Square Error of Approximation) are all above the suggested good or acceptable levels [47], indicating a good model fit. 

## 3. Results

We used hierarchical regression analysis to test all hypotheses. We respectively incorporated control variables, main variables, and interaction variables, and built three models. The F value and R-square value indicated that Model 3 is the best one (see Table 6). 

As shown in Table 6, all three hypotheses are supported. Namely, patient health knowledge seeking behaviors and their social interaction ties in OHCs positively influence a patient’s e-health literacy. Social interaction ties as an environmental factor positively enhance the effect of health knowledge seeking on patient e-health literacy. 

**Figure 2:**
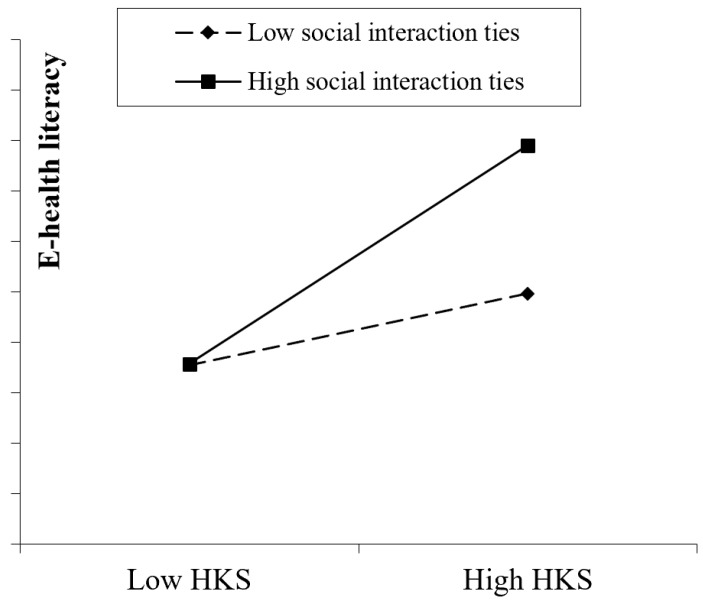
SIT positively moderating the effect of HKS on e-health literacy.

Following the recommended procedures [52], we drew a plot of the moderating effect at one standard deviation below and above the respective means of social interaction ties, health knowledge seeking, and e-health literacy (see Figure 2). As shown in Figure 2, social interaction ties can enhance the effect of health knowledge seeking. When social interaction ties are strong, the effect of patient health knowledge seeking behavior will be high; when social interaction ties are weak, the effect of patient health knowledge seeking behavior will be low.

## 4. Discussion

### 4.1. Theoretical Contributions

This paper makes two significant theoretical contributions. First, we contribute to the literature on the impacts of OHCs on personal health outcomes by exploring the mechanism of how OHC use influences patient e-health literacy. Although OHCs are increasingly popular in public health education and e-health literacy promotion, prior studies are mainly on the impact of the Internet, and few studies have investigated the impact of OHCs [26,27,28,31,32]. In order to address the above gap, this paper explored how behavioral factors (i.e., health knowledge seeking) and social environmental factors (i.e., social interaction ties) influence patient e-health literacy in OHCs. The empirical results show that both health knowledge seeking and social interaction ties positively influence patient e-health literacy; in addition, social interaction ties positively moderate the effect of health knowledge seeking. These findings can enhance our understanding of the mechanism of how OHC use influences patient e-health literacy.

Second, we contribute to the e-health literacy literature by verifying the impact of social environmental factors. Although many studies have explored the antecedents of e-health literacy, they mainly focus on the impacts of demographic factors (e.g., age, gender, and education) [2,26,28,30,31]. Scholars appeal for an emphasis on the importance of accessible media environments and contextual spheres [34]. This study has addressed the above appeal and verified the direct and moderating effects of a social environmental factor (i.e., social interaction ties) on e-health literacy promotion in OHCs. This finding enriches the literature on the roles of social environmental factors in e-health literacy promotion.

### 4.2. Practical Implications

This paper verified the roles of OHCs in promoting e-health literacy and has two significant practical implications in terms of public health promotion. First, considering that this paper has provided positive evidence supporting the role of OHCs in e-health literacy promotion, health caregivers and educators can use OHCs to conduct e-health literacy education. They can organize health promotion courses via OHCs and deliver more health knowledge to vulnerable groups. 

Second, OHC managers should form policies to encourage health knowledge contribution and social networking activities. Considering the positive roles of health knowledge seeking behaviors and social interaction ties, OHC managers can encourage different health-related stakeholders to contribute more health knowledge and member interactions. These endeavors are helpful to keeping OHCs vibrant.

## 5. Conclusions

This paper focused on the factors influencing patient e-health literacy in OHCs. We identified one behavioral factor and one social environmental factor and developed a model for patient e-health literacy based on the SCT and prior studies on e-health literacy. Our empirical results based on 333 valid data points provided two crucial findings: First, both health knowledge seeking and social interaction ties positively influence patient e-health literacy in OHCs. Second, social interaction ties positively moderate the effect of health knowledge seeking on patient e-health literacy.

This paper has three potential limitations that might undermine our findings. First, we treated patient health knowledge seeking as one integral construct and did not explore the effect of behavior frequencies. Future studies could link patient actual health knowledge seeking behaviors and their e-health literacy together. Second, we did not differentiate the types of health knowledge. Different health knowledge might have different effects on e-health literacy [2]. Future studies could take this into consideration. Third, the time-ordering effect between OHC participation behavior and e-health literacy might be a problem. We had tried to control the above effect by setting OHC participation behavior as time perfect tense while setting e-health literacy as simple present tense. Future studies can also focus on the factors influencing patients’ OHC participation behaviors.

## Figures and Tables

**Table 1:** A summary of prior studies on e-health literacy.

Sources	Context/Objective	Independent Variables	Dependent Variables	Findings
[26]	Internet, 2371 parents	e-health literacy	parent’s gender; parent’s race/ethnicity; parental language spoken at home; parent’s educational attainment; parent’s marital status; household type; child’s health; age	Exception of parent’s gender, parent’s marital status, and household type, all other factors have positive effects
[27]	Internet, 182 middle schoolers	e-health literacy	outcome expectations; training involvement; health motivation; perceived injunctive norm; perceived descriptive norm; subjective norm; personal norm	Exception of health motivation, all other factors have positive effects
[28]	59 college students	levels of e-health literacy	race, age, class standing, college major, final course grades, use of the Internet, time spent on the Internet	Only the effect of use of the Internet is significant and positive
[29]	525 valid college students	e-health literacy (as a mediator)	health status; degree of health concern	All effects are significant and positive
[30]	83 lung cancer survivors	e-health literacy	age; gender; living situation; overall health; overall quality of life; histology; education; access to e-resources	Only the effects of education and access to e-resources are significant and positive
[31]	1917 parents and 1417 students	e-health literacy	parent: age; education; marital status; household poverty; area; parent Internet skill confidence; parent Internet skills adolescent: sex; grade; academic performance; adolescent health information literacy	Parent: Exception of age, marital status, and area, all other factors have positive effects Adolescent: Exception of sex, all other factors have positive effects
[32]	192 participants	e-health literacy	gender; department; education level; health status; monthly income; website preference categories	All effects are positive and significant
[33]	65 traditional college students and 143 older adult students	overall e-health literacy; functional e-health literacy	age	Age difference does exist between different groups
[2]	1162 patients who use the Internet	e-health literacy	age; self-rated health; Internet use frequency; online health information seeking frequency; types of health information sought	Age difference exists. All other effects are positive and significant

**Table 2:** Descriptive statistics (*n* = 333).

Variables	*n*	%
Gender		
Male	112	33.6
Female	221	66.4
Age (years)		
< 16	1	0.3
16–25	86	25.8
26–35	82	24.6
36–45	69	20.7
46–55	66	19.8
> 55	29	8.7
Education		
High school and below	80	24
College	73	21.9
Bachelor	110	33
Master and above	70	21
City		
First tier	112	33.6
Second tier	113	33.9
Others	108	32.4
Prior Internet Experience (years)		
< 1	14	4.2
1–2	17	5.1
2–3	32	9.6
3–4	132	39.6
4–5	5	1.5
> 5	133	39.9
Tenure (years)		
< 1	139	41.7
1–2	106	31.8
2–3	44	13.2
3–4	24	7.2
4–5	3	0.9
> 5	17	5.1

**Table 3:** Items and Factor Loadings.

Constructs	Items	HKS	SIT	EHL
Health knowledge seeking	I often use this online health community (OHC) to seek knowledge	0.817	0.140	0.145
	I frequently use this OHC to seek knowledge	0.873	0.252	0.152
	I spend a lot of time using this OHC to seek knowledge	0.773	0.319	0.071
Social interaction ties	I maintain close social relationships with some members in this OHC	0.277	0.844	0.055
	I spend a lot of time interacting with some members in this OHC	0.295	0.840	0.091
	I know some members in this OHC on a personal level	0.179	0.879	0.118
	I have frequent communication with some members in this OHC	0.130	0.926	0.109
E-health literacy	I know how to find helpful health resources on the Internet	0.179	0.010	0.795
	I know how to use the Internet to answer my health questions	0.181	0.023	0.841
	I know what health resources are available on the Internet	0.183	0.036	0.852
	I know where to find helpful health resources on the Internet	0.177	0.102	0.831
	I know how to use the health information I find on the Internet to help me	0.158	0.139	0.825
	I have the skills I need to evaluate the health resources I find on the Internet	0.067	0.169	0.780
	I can tell high-quality from low-quality health resources on the Internet	0.044	0.157	0.781
	I feel confident in using information from the Internet to make health decisions	0.101	0.201	0.768
Cronbach’s α		0.835	0.928	0.933
C.R.		0.825	0.929	0.931
AVE		0.662	0.767	0.630

Note: HKS, SIT, and EHL respectively short for health knowledge seeking, social interaction ties, and e-health literacy; C.R. is short for composite reliability; AVE is short for average variance extraction.

**Table 4:** Covariance Matrix.

Variables	Mean	SD	Gender	Age	Edu.	City	Tenure	PIE	HKS	SIT	EHL
Gender	1.660	0.473	-								
Age	3.600	1.303	-0.018	-							
Edu.	2.510	1.074	−0.242 **	−0.282 **	-						
City	2.010	0.814	−0.060	−0.027	0.368 **	-					
Tenure	4.490	1.435	−0.149 **	0.098	0.023	−0.038	-				
PIE	2.090	1.316	−0.289 **	−0.333 **	0.553 **	0.235 **	0.280 **	-			
HKS	3.382	0.945	0.084	0.250 **	−0.250 **	−0.337 **	−0.084	-0.154 **	0.813		
SIT	2.728	1.020	0.038	0.204 **	−0.335 **	−0.305 **	0.001	−0.236 **	0.502 **	0.876	
EHL	3.705	0.761	0.011	0.081	−0.030	−0.055	−0.033	0.007	0.328 **	0.261 **	0.793

Note: ** *p* < 0.01; Edu. and PIE are short for education and prior Internet experience, respectively.

**Table 5:** Fit Indices.

Indices	χ2	df	χ2/df	GFI	AGFI	NFI	CFI	RMSEA
Results	229.840	84	2.736	0.915	0.878	0.943	0.963	0.072
Criteria	-	-	< 3	> 0.9	> 0.8	> 0.9	> 0.9	< 0.08

**Table 6:** Results of hierarchical regression.

	Model 1		Model 2		Model 3		VIF
	β	T Value	β	T Value	β	T Value	
Control variables							
Gender	0.002 ns	0.034	0.012 ns	0.212	0.002 ns	0.041	1.143
Age	0.078 ns	1.273	0.018 ns	0.302	0.034 ns	0.590	1.269
Edu.	0.001 ns	0.017	0.046 ns	0.675	0.041 ns	0.609	1.706
City	0.013 ns	0.213	0.056 ns	0.993	0.061 ns	1.087	1.204
Tenure	−0.034 ns	−0.564	−0.028 ns	−0.496	−0.017 ns	−0.308	1.183
PIE	−0.023 ns	−0.305	0.069 ns	0.969	0.058 ns	0.820	1.884
Main variables							
HKS			0.280 ***	4.525	0.288 ***	4.697	1.438
SIT			0.166 **	2.649	0.148 *	2.380	1.475
Interaction variables							
SIT × HKS					0.146 **	2.787	1.041
R^2^	0.009		0.133		0.153		
Adjusted R^2^	−0.009		0.111		0.130		
△R^2^	0.009		0.124		0.020		
F (df)	0.479 (6) ns		6.206 (8) ***		6.495 (9) ***		
△F	0.479 ns		23.192 ***		7.769 **		

Note: * *p* < 0.05, ** *p* < 0.01, *** *p* < 0.001, ns, nonsignificant.
